# A novel mutation in the *GARS* gene in a Malian family with Charcot‐Marie‐Tooth disease

**DOI:** 10.1002/mgg3.782

**Published:** 2019-06-07

**Authors:** Abdoulaye Yalcouyé, Seybou H. Diallo, Thomas Coulibaly, Lassana Cissé, Salimata Diallo, Oumar Samassékou, Salimata Diarra, Dramane Coulibaly, Mohamed Keita, Cheick O. Guinto, Kenneth Fischbeck, Guida Landouré, The H3Africa Consortium

**Affiliations:** ^1^ Faculté de Médecine et d'Odontostomatologie USTTB Bamako Mali; ^2^ Service de Neurologie, Centre Hospitalier Universitaire Gabriel Touré Bamako Mali; ^3^ Service de Neurologie, Centre Hospitalier Universitaire du Point “G” Bamako Mali; ^4^ Neurogenetics Branch, National Institutes of Neurological Disorders and Stroke Bethesda MD; ^5^ Service de Médecine, Centre Hospitalier Universitaire Mère‐Enfant le "Luxembourg" Bamako Mali; ^6^ Service d'ORL, Centre Hospitalier Universitaire Gabriel Touré Bamako Mali

**Keywords:** CMT, CMT2D, *GARS*, Mali, novel mutation

## Abstract

**Background:**

Charcot‐Marie‐Tooth (CMT) disease is a very heterogeneous neurological condition with more than 90 reported genetic entities. It is the most common inherited peripheral neuropathy; however, cases are rarely reported in sub‐Saharan Africa. In addition, only few families, mostly of Caucasian ancestry, have been reported to have Charcot‐Marie‐Tooth disease type 2D (CMT2D) mutations. To date no case of CMT2D was reported in Africa. We present here a consanguineous family with CMT phenotype in which a novel mutation in the *GARS* (glycyl‐tRNA synthetase) gene was identified.

**Methods:**

Patients were examined thoroughly and nerve conduction studies (NCS) were performed. DNA from the proband was used for CMT gene panel testing (including 50 genes, *PMP22* duplication and *mtDNA*). Putative mutations were verified in all available family members to check for segregation.

**Results:**

Two individuals, a male and a female, were found to be affected. Symptoms started in their teenage years with muscle weakness and atrophy in hands. Later, distal involvement of the lower limbs was noticed. Patients complained of minor sensory impairment. NCS showed no response in the upper as well as the lower limbs. Genetic testing surprisingly identified a novel heterozygous missense mutation c.794C>A (p.Ser265Tyr) in the *GARS* gene associated with CMT2D. This variant segregated with the disease in the family and was also seen in the mother who presented no symptoms.

**Conclusion:**

This is the first report of a genetically confirmed CMT2D case in Africa, expanding its genetic epidemiology. Increasing access to genetic testing may reveal more novel CMT variants or genes in the African population that could be relevant to other populations and further our understanding of their mechanism.

## 1 INTRODUCTION

Charcot‐Marie‐Tooth (CMT) disease also called hereditary motor and sensory neuropathy is a heterogeneous group of degenerative peripheral nerve disorders. It is characterized by a progressive demyelination or axonal degeneration and cell death resulting in distal muscle weakness and atrophy and sensory loss. Charcot‐Marie‐Tooth disease type 2D (CMT2D) is a classic axonal peripheral sensorimotor neuropathy characterized by weakness and atrophy of more upper than lower limbs involving mostly thenar and first dorsal interosseous muscles. Earliest symptoms in many individuals include transient cramps and pain in hands and calf muscles on exposure to cold or exertion. Sensory loss may affect all modes and can be variable within and across families. The disease is caused by mutations in the glycyl‐tRNA synthetase (*GARS*, OMIM 601472) gene (Antonellis et al., 2003) and distal spinal muscular atrophy type V (dSMA‐V) which does not present sensory impairment is an allelic disorder (Sivakumar et al., 2005).

Although described in other populations, no genetically confirmed CMT2D case has been reported in the literature in Africa in general and West Africa in particular. In this study, we report a novel mutation in *GARS* that causes autosomal dominant CMT2D in a Malian family.

## 2 METHODS

### 2.1 Ethical compliance

This study was approved by the Ethics Committee of the Faculty of Medicine and Dentistry, University of Sciences, Techniques and Technologies of Bamako, Mali.

### 2.2 Clinical and genetic analysis

All of the individuals included in the study were evaluated by a group of neurologists after giving consent. Blood chemistries including glucose and vitamin B12 levels were done to exclude common acquired causes of polyneuropathy. Nerve conduction studies (NCS), ENT and ophthalmologic examinations were done to assess peripheral nerve, ear, or ocular involvement. DNA was extracted from peripheral blood in all available family members for genetic analysis. *PMP22* duplication/deletion (GenBank accession number: NG_007949.1) analysis was done first, and then a Next‐generation CMT gene panel testing composed of 50 genes and the *mtDNA* (Medical Neurogenetics, Atlanta, GA) including *GARS* gene (GenBank accession number: NG_007942.1) was performed on the index patient. The putative mutation was verified in SNP databases to exclude rare variants, and was checked in all available family members. In silico analyses with PolyPhen, I‐Mutant and SIFT were performed for deleteriousness.

## 3 RESULTS

**Figure 1:**
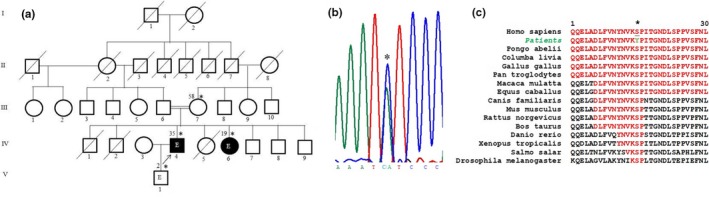
Pedigree of the family with CMT2D and genetic data. (a) Pedigree of the family with CMT2D. Asterisks represent individuals seen in clinic, numbers on left are ages at diagnosis, and the arrow shows the proband. The symbol “E” stands for epilepsy. (b) represents the chromatogram showing the “C” to “A” change (asterisk), and (c) a portion of the highly conserved core catalytic domain (red) where the mutated serine residue is highlighted and conserved from humans to flies (asterisk)

The patients are from a consanguineous family of Bambara ethnicity, and two out of the five living siblings presented with distal muscle weakness and atrophy (Figure 1a). Family history showed that neither of the parents reported symptoms. Symptoms started for both patients when they were around 12 years old with upper limb muscle weakness that progressed over time and involved the thenar and interosseous muscles. Later, lower limbs were also involved. On examination, both affected individuals had distal muscle weakness and atrophy and sensory loss (pin‐prick and vibration) more pronounced in the upper than the lower limbs, decreased to absent reflexes, and plantar stimulation was neutral. Overall, symptoms were worse in the older brother who had claw hands, and both had steppage gait. Past medical history is consistent with recurrent seizures in both patients around age 12, and EEG has showed slow frontal temporal waves in the older patient, though he has no active seizures at present. NCS showed no response in any of the nerves tested. The clinical and laboratory findings are summarized in Table 1. Genetic testing of the proband identified a heterozygous missense variant in the *GARS* gene at position c.794C>A, leading to the amino acid change Ser265Tyr (Figure 1b). Sequencing of DNA from other family members showed that the affected sister carried the Ser265Tyr variant as well as the mother who had no obvious symptoms. Thus, what appeared to be autosomal recessive inheritance was instead autosomal dominant with variable penetrance. The Ser265 residue is conserved across a wide range of species and is located in a highly conserved domain of the protein (Figure 1c). In addition, the c.794C>A change was not found in SNP databases (ExAC Browser, ClinVar, dbSNP, 1000genome), and was shown deleterious in silico.

**Table 1:** Phenotypic characteristics of subjects with CMT2D

Patient	Clinical and demographic features							Nerve Conduction Studies						
	Age (year)	Sex	Age of onset (year)	First symptom	Distal upper limb weakness and atrophy	Distal lower limb weakness and atrophy	Sensory loss	Left peroneal		Sural	Median		Tibial	
								CMAP Amp (mV)	CV m/s	SNAP Amp	CMAP Amp (mV)	CV m/s	CMAP Amp (mV)	CV m/s
IV.4	35	M	12	Hand weakness	Severe	Severe	Moderate	NR	NR	NR	NR	NR	NR	NR
IV.6	19	F	10	Walking difficulty	Moderate	Moderate	Moderate	NR	NR	NR	NR	NR	NR	NR
III.7	58	F	N/A	N/A	No	No	No	ND	ND	ND	ND	ND	ND	ND

Note

Normal median CMAP >4.5 mV (recorded at abductor pollicis brevis muscle), normal peroneal CMAP >2.5 mV (recorded at extensor digitorus brevis muscle), normal tibial CMAP >6 mV (recorded at abductor hallucis muscle), normal sural SNAP >10 μV, normal F wave <55 ms lower limbs and <32 ms upper limbs.

Amp, amplitude; CMAP, compound motor action potential; CMT2D, Charcot‐Marie‐Tooth disease type 2D; CV, conduction velocity; ND, not done; NR, no response; SNAP, sensory nerve action potential

## 4 DISCUSSION

Charcot‐Marie‐Tooth disease type 2D caused by mutations in the *GARS* gene have been found in a relatively small number of families from different populations in Europe, North America, and Asia (Liao et al., 2015). Mutations in the *GARS* gene have also been shown to cause dSMA‐V, an early onset of pure motor neuropathy. While no CMT2D case was reported in Africa, dSMA‐V has been reported in a family with North African origin (Dubourg et al., 2006).

We report here a novel mutation in *GARS* causing CMT2D in a Malian family. CMT2D is an autosomal dominant disease with high intrafamily variability, and most of the patients present symptoms during the second decade (Sivakumar et al., 2005). The hallmark of the disease is the presenting manifestations in the hands with weakness and atrophy in the thenar and the first dorsal interosseous muscles and the sparing of the hypothenar eminence, which is involved later in the disease course. Lower limbs are involved in about half of affected individuals, and mild loss of vibration sense is observed in a third of individuals in the late stage of the disease. High intra‐ and interfamilial variability is observed in this disease. This is reflected in the family we report here in which two siblings presented with symptoms around 12 years of age and neither of the parents complained of symptoms. The patients’ mother was tested positive for the variant, but her neurological examination was normal. Unfortunately, she was not available for NCS as it has been shown that in CMT conduction abnormalities may precede clinical ones. The adolescent onset and the presence of sensory symptoms in the patients indicate that they present CMT2D rather than dSMA‐V. The underlying mechanism distinguishing CMT2D from its allelic disease dSMA‐V is still not well elucidated although a recent study has suggested that CMT2D pathogenesis involves both neurodevelopmental and neurodegenerative processes (Sleigh et al., 2017).

Glycyl‐tRNA synthetase is a member of the aminoacyl‐tRNA synthetase family. The human GARS protein has three major functional domains including the core catalytic domain located at the 92nd–168th residues and the 241st–324th residues where the mutation reported in this study is located (Rohkamm et al., 2007). Its function is to catalyze the esterification reaction between the carboxyl group of glycine and its cognate tRNAs, resulting in aminoacylation of the tRNAs, substrates for protein synthesis in the ribosome (Ibba & Soll, 2000). Mutations in or close to the catalytic domain have been previously reported, and reduced GARS catalytic activity or other functional impairment of GARS have been suggested as a cause of the axonal neuropathy (Sivakumar et al., 2005). However, other studies have suggested that toxicity of mutant GARS may cause the neuropathy (Motley et al., 2011).

Some *GARS* mutations have weak genetic and functional evidence of causing CMT (Griffin et al., 2014). However, a mutation of the Ser265 residue leading to a different amino acid change was reported in a family with dSMA‐V (Lee et al., 2012). This, in addition to the conservation of the Ser265 throughout wide range of species, the deleteriousness of the Ser265Tyr change by in‐silico predictions and its absence in SNP databases, suggests that the mutation we report is indeed pathogenic.

We report here the first CMT2D cases in Africa and a novel mutation in *GARS*, expanding the genetic epidemiology of this CMT subtype. The Ser265Tyr variant, which was not previously reported elsewhere, adds to the limited number of *GARS* disease‐associated variants. Although some clinical variability has been published in CMT2D families, the clinical pattern seen in the family presented in this study has rarely been reported. This can be stochastic or due to other genetic or environmental modifiers. Larger cohort studies in Africa will allow phenotype‐genotype studies to understand the phenotypic variability and the underlying mechanism distinguishing CMT2D from dSMA‐V. Such studies may also uncover other new CMT variants or genes that can be studied in other populations.

## CONFLICT OF INTEREST

The authors declare that they have no conflict of interest.
